# A cost-analysis of complex workplace nutrition education and environmental dietary modification interventions

**DOI:** 10.1186/s12889-016-3988-7

**Published:** 2017-01-09

**Authors:** Sarah Fitzgerald, Ann Kirby, Aileen Murphy, Fiona Geaney, Ivan J. Perry

**Affiliations:** 1 Department of Epidemiology and Public Health, University College Cork, 4th Floor, Western Gateway Building, Western Road, Cork, Ireland; 2 Department of Economics, Aras na Laoi, University College Cork, Cork, Ireland

**Keywords:** Cost-analysis, Micro-costing, Workplace dietary interventions, Environmental dietary modification, Workplace health promotion

## Abstract

**Background:**

The workplace has been identified as a priority setting to positively influence individuals’ dietary behaviours. However, a dearth of evidence exists regarding the costs of implementing and delivering workplace dietary interventions. This study aimed to conduct a cost-analysis of workplace nutrition education and environmental dietary modification interventions from an employer’s perspective.

**Methods:**

Cost data were obtained from a workplace dietary intervention trial, the Food Choice at Work Study. Micro-costing methods estimated costs associated with implementing and delivering the interventions for 1 year in four multinational manufacturing workplaces in Cork, Ireland. The workplaces were allocated to one of the following groups: control, nutrition education alone, environmental dietary modification alone and nutrition education and environmental dietary modification combined. A total of 850 employees were recruited across the four workplaces. For comparison purposes, total costs were standardised for 500 employees per workplace.

**Results:**

The combined intervention reported the highest total costs of €31,108. The nutrition education intervention reported total costs of €28,529. Total costs for the environmental dietary modification intervention were €3689. Total costs for the control workplace were zero. The average annual cost per employee was; combined intervention: €62, nutrition education: €57, environmental modification: €7 and control: €0. Nutritionist’s time was the main cost contributor across all interventions, (ranging from 53 to 75% of total costs).

**Conclusions:**

Within multi-component interventions, the relative cost of implementing and delivering nutrition education elements is high compared to environmental modification strategies. A workplace environmental modification strategy added marginal additional cost, relative to the control. Findings will inform employers and public health policy-makers regarding the economic feasibility of implementing and scaling dietary interventions.

**Trial registration:**

Current Controlled Trials: ISRCTN35108237. Date of registration: The trial was retrospectively registered on 02/07/2013.

## Background

Chronic diet-related diseases such as obesity, cardiovascular disease, type 2 diabetes and stroke continue to endanger population health worldwide [1, 2]. The full impact of diet-related diseases extends beyond the population health burden to include a considerable financial burden which is attributable to escalating healthcare spending [3–6]. This financial burden is borne not only by society but also by employers as diet-related diseases have been linked to absenteeism and productivity loss in the workplace [7]. In this environment of mounting healthcare costs and on-going financial constraints, emphasis is being increasingly placed on treatment of diet-related diseases rather than preventative measures [2]. Consuming an unhealthy diet has been identified as one of the main modifiable behavioural risk factors for the development of chronic diet-related diseases [2]. Dietary interventions that support low intakes of saturated fat, sugar and salt and high intakes of fruit and vegetables are considered to be one of the preferred cost-effective interventions for easing the burden [1–3].

The surrounding environment in which an individual lives and works has the potential to influence their health-related behaviour. It is widely accepted that modification of these surrounding environments can promote behaviour change at a population level [8]. The workplace environment has been identified as a priority setting for the promotion of healthy dietary behaviours given that individuals are now spending up to two-thirds of their waking hours in their workplace [5, 6, 9, 10]. Workplaces can facilitate the delivery and implementation of health promotion interventions by providing the necessary infrastructure and access to a stable population within a controlled environment [5, 9].

However, uncertainty exists with regards to the effectiveness of workplace dietary interventions [11, 12]. Previous dietary interventions have demonstrated limited efficacy with small effect sizes [11, 13–15]. There is some evidence to suggest that workplace nutrition education interventions can have modest positive effects on dietary behaviour in terms of intakes of fruit and vegetables [11, 12]. However, there is a dearth of evidence regarding workplace dietary modification interventions. Furthermore, the evidence base regarding the costs associated with workplace dietary interventions is extremely limited as many interventions have failed to report cost data alongside effectiveness data [11, 15]. Previous literature suggests that there is a need to accurately determine the associated costs of workplace interventions in order to reliably determine cost-effectiveness [15]. Workplace dietary interventions have become a focal point on organisational agendas in an effort to reduce the costs associated with absenteeism and productivity losses however, the cost to employers associated with implementing and delivering these interventions remains largely unknown [10, 16].

The Food Choice at Work Study (FCW) was a complex workplace dietary intervention trial which assessed the comparative effectiveness of an environmental dietary modification intervention and a nutrition education intervention both alone and in combination versus a control workplace [17]. This study will inform a future economic analysis of the FCW interventions. As previously outlined, the financial impact associated with workplace dietary interventions have been poorly documented in the evidence to date. Hence, there is a need for a detailed exposition of costs for each intervention to be presented. The aim of this study was to provide a transparent assessment of the costs associated with implementing and delivering the FCW interventions over a 1-year period. As the costs of implementing workplace interventions are usually borne by employers, the cost-analysis was conducted from the perspective of the employer and the intervention costs were measured against a control workplace. Findings provide a novel insight into the costs associated with workplace dietary interventions. Furthermore, the study delivers accurate cost data which will assist both employers and public health policy makers in making evidence-based decisions regarding the economic feasibility of implementing dietary interventions and also on their potential scalability.

## Methods

### Data source

A detailed description of the study design, intervention elements and methods of the FCW study has been published previously [17]. In summary, a cluster controlled trial was conducted in four large multi-national manufacturing workplaces in Cork, Ireland over a 9-month period. A comprehensive list of Cork based manufacturing workplaces were obtained from the Irish Industrial Development Authority (IDA) website and were systematically screened for eligibility. Workplaces were deemed eligible to participate if they employed >250 employees, were located in Cork, had a daily workplace canteen and were able to commit to the intervention for the duration of the study. Four workplaces were purposively selected and allocated to the interventions accordingly. Employees were selected using random number generation software (Microsoft Excel) and were invited to participate if deemed eligible. Eligible employees were permanent, full-time employees who purchased and consumed at least one daily meal in their workplace. Employees were excluded if they did not work full-time, travelled regularly for work, were medically advised not to participate, were on long-term leave or were involved in an on-going diet programme external to their workplace. Further detail on workplace and employee recruitment has been published elsewhere [17].

### Interventions

Participants in all workplaces, underwent physical assessments (height, weight, midway waist circumference and resting blood pressure measurements) and 24-h dietary recalls which were conducted by trained research assistants/nutritionists as per the Standard Operating Procedures (SOP) manual for the FCW study [18]. In the control workplace data was collected at baseline and at each stage of follow-up with participants informed that they were involved in a university-led study designed to observe employees’ dietary behaviours. Employees in this workplace also underwent physical >assessments which incurred costs for both employee and nutritionist time. However, as this was the control, nutrition education was not provided and no modifications were made to the environment. A nutrition education intervention was provided in the second workplace, an environmental dietary modification intervention was implemented in the third workplace and the fourth workplace received a combined intervention (both nutrition education and environmental dietary modification interventions). Table 1 contains a detailed description of the multicomponent interventions elements.

**Table 1:** Description of the intervention elements

Intervention	Description of elements
Nutrition education	*Group presentations:* Monthly nutrition sessions (30 min per session) were delivered in the workplace by the FCW nutritionist. Topics included portion control, food labelling and general healthy eating guidelines. Sessions were repeated to ensure they were accessible to employees on different shift cycles.
	*Individual nutrition consultations:* Participants received one-to-one dietary counselling with the FCW nutritionist. Consultations were tailored for each participant based on their lifestyle, physical assessment results and dietary recall assessments. The nutritionist provided advice on how to follow a healthy diet, reach/maintain a healthy body weight and achieve healthy resting blood pressure. Participants also received a healthy eating booklet and a personalised measurement card.
	*Detailed nutrition information:* Detailed nutrition information was prepared by the FCW nutritionist and displayed in the workplace throughout the intervention. The information included posters, leaflets, emails and daily calorie menu labelling with a unique traffic-light coding system. A healthy eating chat table was also provided twice a month during break times to provide employees with an opportunity to ask the nutritionist about healthy eating.
Environmental dietary modification	*Menu modification:* Saturated fat, sugar and salt were restricted. Stock and bouillon were replaced with low-salt stock options. Salt was replaced with fresh herbs, spices and garlic for additional flavour. High salt savoury options, high-salt products and processed meats were reduced and replaced where possible with low-salt options. Full-fat dairy products were replaced with low-fat options where possible. Cream and cheese were not used as a garnish on meals and the amount of cheddar in all meals was reduced. Cooking methods using oil (deep-fat frying) were limited and replaced with boiling, poaching, grilling, baking and steaming where possible. Plant oils were introduced for cooking. Sauces and accompaniments were not added to any meal unless requested by the employee. Chips and French fries were removed from the menus 2 days a week and were replaced with different potato options such as baked potatoes. Soft carbonated drinks were restricted and replaced with water, milk and unsweetened options.
	*Increase in fibre and availability of fruit and vegetables:* White pasta, rice and bread were replaced with wholegrain alternatives. Fruit and vegetables were added to rice, pasta, soup and meat dishes. Fresh whole fruit was made available throughout the day and a buffet-style fresh salad bar was available to accompany any dish on a daily basis.
	*Price discounts:* Portions of whole fresh fruit were offered at discount prices on a daily basis within the confines of the pre-existing catering contract.
	*Strategic positioning of food:* Healthier alternatives were strategically positioned throughout the workplace canteen. Healthy snacks, such as fresh fruit, dried nuts, seeds, brown sandwiches and brown soda bread were positioned at eye level at the entrance of the canteen and in the vending machines. Free-flowing salt and sugar were removed from tables and replaced with sachets.
	*Portion size control:* Standard serving tools were used to control portion size at mealtimes. Catering staff received training from the FCW nutritionist regarding strict portion size control.

The complex interventions were guided by a soft paternalistic ‘nudge’ theoretical perspective [19]. The nutrition education intervention was designed to create positive reinforcement with indirect suggestions for healthy food choices in an effort to improve the employees’ dietary behaviours. Elements such as the one-to-one nutrition consultations and calorie and traffic light menu labelling were designed to prompt conscious consideration of food choices. The environmental dietary modification interventions were guided by choice architecture [19]. These elements were designed to prompt both conscious (repositioning of healthier alternatives) and unconscious (menu modification) thoughts. The intervention design was developed by the FCW research team (nutritionists/dietician) and advised by catering stakeholders (Catering Managers Association of Ireland (CMAI)). The research team collaborated with the workplace stakeholders (occupational health and catering managers) to implement the FCW interventions within each individual workplace. The FCW nutritionist provided training to catering staff with regards to compliance with the recommended menu modifications and portion size control, specifically for the environmental dietary modification intervention. Each workplace was assigned a research workplace leader (nutritionist) who collaborated with workplace stakeholders to co-ordinate data collection for rotating shift schedules and monitor adherence to all elements of intervention. Ethical approval was granted to the FCW study by the Clinical Research Ethics Committee of the Cork Teaching Hospitals in Ireland in March 2013.

### Measuring intervention costs

All costs incurred from implementing and delivering the FCW interventions over a 1-year period were measured from the perspective of the employers. A total of 850 employees took part in the FCW study (Control: *N* = 111, Education: *N* = 226, Environment: *N* = 113 and Combined: *N* = 400). The number of employees recruited per workplace was proportionate to the company size. Costs were initially measured for 850 employees, however for the purposes of this study, total costs were standardised for a cohort of 500 employees per workplace. This was conducted for ease of comparison as having the same sample size per workplace will allow employers to use the costs as a benchmark in similarly sized workplaces that are implementing dietary interventions. Similarly, standardising costs over a 1-year period rather than a 9-month period (duration of the intervention) will further increase the comparability of findings for employers.

In order to obtain an accurate estimate of costs associated with implementing and delivering the intervention outside of a trial setting, research costs (equipment used for carrying out physical assessments and cost of analysing data) were excluded from the analysis. Furthermore, the costs incurred through conducting physical assessments were excluded from the total costs but are presented in the results section of this study. A bottom-up approach, using micro-costing was employed to disaggregate the cost of each intervention. The resources consumed in each intervention were identified and the unit costs of the resources were multiplied by the quantities used [20]. The FCW research team who were involved in the implementation of the interventions identified each intervention pathway and thus, each of the cost components to be measured. This identification occurred through a combination of interviews with the FCW nutritionists, research assistants and employees and through referencing of workplace diaries that were kept by each workplace leader. Labour and material costs were the only two types of costs to be identified. These costs were subsequently separated into two main phases, the first of which represented set-up costs (costs incurred through intervention implementation). These set-up costs were modest and were therefore not annualised (Table 3). The second phase of costs represented maintenance costs (costs incurred through intervention delivery). The costs incurred through employees undergoing physical assessments with research assistants were also identified and are presented separate to the main estimates. Five cost categories were identified for each phase:

1. *Nutritionist costs:* Staff costs for the nutritionist included the time it took for; food product and menu analysis; application of calorie and traffic light coding to menus; training of catering staff with regards to intervention compliance, preparation of detailed dietary information; individual nutrition consultations; group presentations; healthy eating chat tables and monitoring adherence to interventions.
2. *Catering costs:* This category consisted of staff costs for the catering manager, head chef and catering assistants. This included the time associated with discussing and agreeing on menu changes and other intervention elements, the displaying of calories and traffic lights on menus and receiving training from the nutritionist regarding portion size control.
3. *Management stakeholder:* This category included staff costs for workplace staff who were involved in the implementation or delivery of the interventions. Staff costs arose from time spent at meetings between the environmental health and safety manager (representative from occupational health department) and the nutritionist at the outset of the study and meetings that were held to discuss the logistics of the monthly group presentations and nutrition consultations.
4. *Employee costs:* These costs included the time associated with employees attending the individual nutrition consultations during working hours and also lost leisure time for employees attending monthly group presentations and the healthy eating chat table during lunch breaks.
5. *Printing and material costs:* This category was comprised of costs related to printing material and menu holders for the display of detailed nutrition information in the workplaces.

Table 2 contains a detailed breakdown of how the resources were identified, measured and valued for each of the cost categories.

**Table 2:** Identification, measurement and valuation of cost categories

	Nutritionist costs			Catering costs			Management stakeholder costs			Employee costs			Printing and material costs		
	# Units (hours)	€/unit	Source	# Units (hours)	€/unit	Source	# Units (hours)	€/unit	Source	# Units (hours)	€/unit	Source	# Units (print materials)	^a^€/unit	Source
Nutrition education only	875	27.50	Interview	HC: 10.0	29.65	Salary scale [24]	EHS: 5.96	51.68	Salary scale [24]	787.2	21.94	National average wage [26]	4048	Range (0.03 to 3.00)	FCW expense reports
				CM: 33.4	44.48										
Environmental modification only	568.8	27.50	Interview	HC:8.1	29.65	Salary scale [24] Job advert	EHS: 5.96	51.68	Salary scale [24]	362.7	21.94	National average wage [26]	254	Range (0.03 to 3.00)	FCW expense reports
				CM: 4.0	44.48										
				CA:4.0	15.63										
Combined intervention	953	27.50	Interview	HC:18.1	29.65	Salary scale [24] Job advert	EHS: 5.96	51.68	Salary scale [24]	787.2	21.94	National average wage [26]	4048	Range (0.03 to 3.00)	FCW expense reports
				CM: 35.1	44.48										
				CA:4.0	15.63										
Control	489.2	27.50	Interview	***-***	***-***	***-***	***-***	***-***	***-***	362.7	21.94	National average wage [26]	***-***	***-***	***-***

#units = number of units used over a 1-year period; €/unit = price per unit; *HC* head chef, *CM* catering manager, *CA* catering assistant, *EHS* environmental health and safety officer

^a^Represents a range of different print materials (leaflets, posters, menu displays, measurement cards, healthy eating guidelines booklet) all valued at a unit price, ranging from €0.03 to €3.00

### Valuation of intervention costs

These resources were valued in monetary terms using standard techniques [20–23]. For each intervention, the total intervention cost was estimated. The primary outcome was the net cost of each intervention (nutrition education, environmental dietary modification and combined) compared to the control workplace. Staff costs for the study nutritionist were estimated based on an hourly rate for a private nutrition consultancy service which was obtained through interview with a nutritionist. Similarly, staff costs for catering assistants were estimated using market prices which were sourced from job advertisements for food service assistant positions with the catering companies who participated in the FCW study. The Department of Health consolidated salary scales were used to estimate staff costs for the catering manager, head chef and environmental health and safety officer [24]. The median point on the scales for a catering manager, a senior chef and a senior environmental health and safety officer were selected as recommended in national guidelines (Health Information and Quality Authority (HIQA)) [23]. In order to adjust for associated non-pay costs, employers PRSI (10.75%) was added to the mid-point of the pay range, the net pension cost (4%) was added to the direct salary cost and overhead costs (25%) were then added to estimate the total staff cost [23]. Hourly costs for each staff category were then subsequently calculated as per the Government Regulatory Impact Analysis (RIA) guidelines [25]. Employee time was valued using the national average wage as specified by the Central Statistics Office (CS0) [26].

For printing and material costs, cost data were obtained from FCW expense reports which were made available by the FCW project manager. With regards to the implementation and delivery of menu modifications, no extra costs were incurred. All menu modifications (which were recommended by the FCW nutritionists) were within the existing budget predefined by the catering provider for that workplace.

## Results

A detailed breakdown of the total costs associated with setting up and implementing the nutrition education intervention, the environmental dietary modification intervention, the combined intervention and the control over a 1-year period for a cohort of 500 employees are contained in Table 3. Across each of the interventions, two principal types of costs were identified; 1) staffing costs (the nutritionist, management stakeholders from the workplaces, catering staff and employees) and 2) printing and material costs. Physical assessment costs were also identified as significant cost across each of the interventions. However, as research costs were excluded from analysis, physical assessment costs are presented separate to the total costs.

**Table 3:** Intervention costs

		Nutrition education Costs (€)	Environmental modification Costs (€)	Combined Costs (€)	Control Costs (€)
Set-up costs	Nutritionist	566	2434	3041	-
	Catering costs	47	480	480	-
	Management stakeholder costs	103	103	103	-
	Printing and materials	1019	85	1019	-
	Employee time	53	53	53	-
	*Sub-total*	*1788*	*3154*	*4696*	*-*
Maintaining costs	Nutritionist	14,487	330	14,157	-
	Catering costs	1736	-	1736	-
	Management stakeholder costs	205	205	205	-
	Printing and materials	282	-	282	-
	Employee time	10,031	-	10,031	-
	*Sub-total*	*26,741*	*535*	*26,412*	*-*
Physical assessments	Nutritionist	9009	12,879	9009	*13,453*
	Employee time	7188	7906	7188	*7959*
	*Sub-total*	*16,197*	*20,785*	*16,197*	*21,412*
	Total cost of intervention (excluding physical assessments)	28,529	3689	31,108	0
	Annual cost per employee (N = 500) excluding physical assessments	57	7	62	-
	Total cost of intervention (including physical assessments)	44,726	24,474	47,305	21,412
	Annual cost per employee (N = 500) including physical assessments	89	49	95	43

For the nutrition education intervention set-up costs were reported at €1788 (6.3% of total costs) and maintenance costs were reported at €26,741 (93.7% of total costs). The total cost of the nutrition education intervention was estimated at €28,529. The average annual cost per employee for implementing and maintaining the nutrition education intervention was estimated at €57.

The environmental dietary modification intervention reported set-up costs of €3154 (85.5% of total costs) and maintenance costs were reported at €535 (14.5% of total costs). Total costs for the environmental dietary modification intervention were estimated at €3689. The average annual cost per employee for implementing and maintaining the environmental dietary modification intervention was estimated at €7.

For the combined intervention set-up costs were reported at €4696 (15% of total costs) and maintenance costs were reported at €26,412 (85% of total costs). Total costs for the combined intervention were estimated at €31,108. The average annual costs per employee for implementing and maintaining the combined intervention was estimated at €62.

In the control workplace set-up costs and maintenance costs were non-existent as no intervention elements were implemented in the workplace. Total costs of €21,412 were reported, of which physical assessment costs accounted for 100%. Thus, the cost per employee in the control workplace was estimated at zero.

Total costs were higher for the nutrition education intervention when compared to the environmental dietary modification intervention. These higher costs were attributable to the delivery of one-to-one dietary counselling which was the main element driving the maintenance costs in the nutrition education intervention. This element which was not provided in the environmental dietary modification intervention, required substantial investments of both the nutritionist and employees’ time. The cost of this element can be observed in the high nutritionist costs (€14,157) and high employee time costs (€10,031) for maintaining the environmental dietary modification. Similarly, the provision of detailed nutrition information and monthly group nutrition sessions incurred additional costs for the nutrition education intervention (€2151 and €2963). These elements included costs associated with printing and materials, nutritionist time and employee time.

The environmental dietary modification intervention reported marginal additional total costs when compared to the control workplace (€3689). These additional costs were associated with the set-up (€3154) and maintenance (€535) costs that were associated with the environmental dietary modification intervention. This included the time associated with nutritionists modifying menus, training of catering staff with regards to portion size control and monitoring adherence to menu and canteen modifications. Similar to the control, the maintenance of the environmental dietary modification intervention did not incur printing and material costs.

It can be observed that for each intervention, the nutritionist was the main contributor to the costs and accounted for the largest proportion of total costs: nutrition education intervention: 53%, environmental dietary modification intervention: 75% and combined intervention: 55%. In the control workplace, the nutritionist and employees did not incur set-up or maintenance costs as no intervention was implemented. In terms of the other staffing costs, employee time accounted for the second highest proportion of costs for each intervention (nutrition education intervention: 35%, environmental dietary modification intervention: 1.4% and combined intervention: 32%). Catering and workplace staffing costs and printing and material costs accounted for marginal proportions of the total costs.

When physical assessment costs were factored into the analysis, total costs increased substantially for each intervention (Table 3). Total costs increased to €44,726 for the nutrition education intervention, €24,474 for the environmental dietary modification intervention and €47,305 for the combined intervention. Total costs in the control workplace consisted exclusively of physical assessment costs (€21,412), as employees underwent physical assessments but did not receive any intervention elements. Physical assessment costs included costs incurred through employees undergoing physical assessments. Employee time and nutritionist time were the two categories of costs that were associated with physical assessment costs. When physical assessment costs are included in the total costs, the average annual cost per employee for implementing and maintaining the interventions increased to €89 for the nutrition education intervention, €49 for the environmental dietary modification intervention, €95 for the combined intervention and €43 for the control.

## Discussion

This study reports the results of a bottom-up costing study of complex workplace nutrition education and dietary modification interventions. As we are entering into an era where workplace health promotion dietary initiatives are garnering increasing attention, it is imperative that a detailed breakdown of the costs associated with these approaches is reported in a transparent manner. To our knowledge, this study is the first detailed cost-analysis of a complex workplace dietary intervention and therefore the findings can be considered novel. The combined intervention was revealed to be the most expensive intervention to implement and deliver (€31,108) and the nutrition education intervention (€28,529) was found to be considerably more expensive than the environmental dietary modification intervention (€3689) to implement and deliver. These findings persisted with the addition of physical assessment costs to total costs with the combined intervention remaining the most expensive intervention. The findings indicate that the implementation and maintenance of environmental dietary modification strategies in the workplace add minimal additional cost to the control when compared to nutrition education strategies.

In the analysis of this study, physical assessment costs were purposively made distinguishable from the other categories of costs. As the FCW study was a research study, physical assessments were conducted in order to measure the clinical effectiveness of the different interventions. However, in ‘real-world’ settings such as workplaces, such outcome data would not need to be collected and the interventions could be implemented and delivered without physical assessments being carried out.

There is limited available evidence to suggest that workplace health promotion interventions that are based on the provision of nutrition information can result in modest improvements in terms of employee dietary behaviour and weight loss [11, 15, 16]. However, despite this limited evidence and relatively modest outcomes, the provision of nutrition information has remained the primary focus of workplace health promotion initiatives [11]. Employers are continuing to invest in nutrition information based workplace interventions that have demonstrated only limited effectiveness [11]. Moreover, due to the lack of detailed cost data on such interventions, these investment decisions are being made without access to accurate cost estimates.

This study has revealed that the implementation and maintenance of environmental dietary modification interventions is less expensive than nutrition education interventions, irrespective of the inclusion of physical assessment costs. These findings begin to address the paucity of evidence regarding the costs associated with the implementation and delivery of environmental dietary modification strategies in the workplace. There is consensus in the literature that an individual’s surrounding environment has significant capacity to influence their health-related behaviour [8]. Altering an individual’s physical and social environment has been identified as one of the most effective ways of reducing the burden of diet-related disease and the main impetus for achieving behaviour change at a population level [27, 28]. This evidence combined with our findings which indicate environmental dietary modification incurs minimal additional cost, suggests that such dietary modification strategies should be considered for implementation in workplaces rather than relying exclusively on traditional nutrition education strategies. If physical assessment costs are excluded from the total cost of the environmental modification intervention, the average annual cost per employee to implement and deliver the intervention is €7. This cost would be considered to be relatively inexpensive when borne by employers in large multinational manufacturing workplaces.

As previously mentioned, workplace health promotion strategies have become prominent features on organisational agendas both nationally [29] and internationally [2]. However, it is important to acknowledge the limited reported effects of these interventions to date. Previous interventions have demonstrated that combined interventions (workplace environmental dietary modification interventions alone and in combination with nutrition education strategies) can result in small increases (≥ half a portion per day) in fruit and vegetable consumption [11]. Further studies have also suggested that nutrition education strategies can have a moderate positive effect on dietary behaviour with decreases of up to 9% in total dietary fat and increases of up to 16% in daily fruit and vegetable intakes being reported [13–15]. With regards to the effect of the interventions on anthropometric measurements, it is difficult to draw conclusions as previous interventions have relied on self-reported measures for health outcomes which increases the risk of bias [15]. Moreover, the reliability of the evidence is further limited due to the low-intensity design and poor methodological quality of previous studies. Many intervention studies have neglected to include suitably matched control groups and have been poorly evaluated.

A recent study conducted by FCW researchers assessed the effectiveness of the high-intensity complex FCW interventions and provides the strongest evidence to date regarding the effects of these types of interventions. The comparative effectiveness of a workplace environmental dietary modification and an educational intervention both alone and in combination was assessed versus a control workplace [30]. It was reported that there were significant positive changes in intakes of saturated fat (−7.0 g/day (SD 17.6)), salt (−1.3 g/day (95% CI: −2.3, −0.3)) and nutrition knowledge (+3.0 (SD 7.6)) between baseline data collection and 7–9 months follow-up between the combined intervention and the control. Furthermore, significant reductions in measured BMI (−1.2 kg/m^2^ (95% CI: −2.385, −0.018)) were also observed in the combined interventions [30]. Effects in the education intervention and environment intervention workplaces were smaller and generally non-significant.

The implementation of workplace ill-health prevention initiatives continue to be highlighted as a potential strategy for employers to improve employee health outcomes and to reduce escalating costs that are arising as a result of ill-health, absenteeism and lost productivity [29]. Specifically, the Health and Safety Authority advocate for the development of a ‘service-delivery model’ that will support workplaces in the implementation of workplace health promotion and well-being programmes [29]. Our findings suggest that environmental dietary modification strategies could serve as one such potential service-delivery model to support and facilitate employers in implementing workplace ill-health prevention and health promotion strategies at a minimal additional cost. The low maintenance costs (€535) for the environmental modification intervention would also suggest that such modifications strategies could be implemented in workplaces on a long-term basis.

A key strength of this study is the use of micro-costing to estimate the costs of each of the interventions. Micro-costing is considered to be the most useful method to use when estimating the cost of a new health technology or intervention. Therefore, a high-level of precision in our cost estimates in the selected workplace settings can be ensured [20–22]. Despite this precision in the cost estimates, it is important to acknowledge that these estimates were derived from specific dietary interventions that were implemented in atypical multinational manufacturing workplaces. Although the purposive selection of workplaces limits the generalisability of the results, the findings provide some guidance on the potential cost of implementing and delivering similar interventions across different workplace settings.

## Conclusions

This study offers a unique insight into the costs associated with both implementing and maintaining a complex workplace nutrition education and an environmental dietary modification intervention from the perspective of the employer. Findings will be used to inform an economic evaluation of the FCW interventions. Due to the level of uncertainty in the evidence regarding the cost of workplace dietary interventions, providing a detailed exposition of the costs was of particular importance. An environmental dietary modification intervention incurs marginal additional costs when compared to the control. Nutrition education interventions and combined interventions are more costly owing to the set-up and maintenance costs associated with the education strategies, demonstrating the need for careful consideration when selecting suitable education elements. Accurate cost data can be used to determine the potential scalability of such workplace dietary interventions and inform evidence-based decisions. It is envisaged that the findings can be used alongside studies investigating the clinical effectiveness of workplace dietary interventions to inform both employers and public health policy makers on how to achieve an appropriate balance between improving employee health outcomes and the economic feasibility of implementing workplace dietary interventions.

## Abbreviations

BMI: Body mass index
CA: Catering assistant
CM: Catering manager
CMAI: Catering Managers Association of Ireland
CSO: Central Statistics Office
EHS: Environmental Health and Safety Officer
FCW: Food Choice at Work Study
HC: Head chef
HIQA: Health information quality authority
IDA: Irish Industrial Development Authority
RIA: Regulatory Impact Analysis
SOP: Standard Operating Procedures
